# 

**Published:** 1920-06-19

**Authors:** 


### Passing Events and Topics.

### Hospital Benefits by House Shortage.

The following story comes from a seaside resort. After considerable delay a lady secured the option of a suitable residence, and, deciding to purchase, paid the required deposit, only, however, to hear from her medical adviser that because of the state of her health other accommodation would be preferable. The house was subsequently sold within twenty-four hours at a profit of £50. The lady, however, not desiring to "profiteer," forwarded a cheque for £25 to her local hospital.

### Footballer as Hospital Secretary.

The recent appointment to the Metropolitan Hospital, writes a correspondent, is probably the first instance where the football world has supplied a hospital secretary, an event which has been brought about by the selection of Mr. G. B. Dale, of Barnsley fame, to fill the vacancy.

### A Gift Worthy of Imitation.

Sir Ernest Palmer, Bart., has presented a brougham to the Royal Hospital and Home for Incurables, Putney, which will enable some of the suffering inmates to take an occasional drive.

Dr. W. J. Williams, of Grange Road, Middlesbrough, left property valued at £13,863. Books from his library are to be selected by his executors for the Welsh National Library at Aberystwyth. After the death of his wife £500 is to be paid to the North Riding Infirmary, Middlesbrough, and the ultimate residue of his estate to University College of North Wales at Bangor.

### Matrons' and Sisters' Appointments.

ADDENBROOKE'S HOSPITAL, CAMBRIDGE.—Miss C. Kartle and Miss L. Folliott have been appointed sisters. Miss Kartle was trained at Middlesex Hospital, where she was later ward, theatre, and night sister. She has since been sister at St. Mary's Home, Broadstairs. Miss Folliott was trained at St. Thomas's Hospital, and has since been sister at Lord Mayor Treloar's Hospital, Alton. She has also done private nursing.

BIERLEY HALL SANATORIUM, BRADFORD.—Miss Annie Deans has been appointed sister. She was trained at Birmingham Infirmary, and has since been nurse-in-charge of King Edward VII. Sanatorium, Midhurst, and sister at Middleton-in-Wharfedale Sanatorium, Ilkley, and at the Royal National Sanatorium, Bournemouth.

EAST SUFFOLK AND IPSWICH HOSPITAL, BARTLET CONVALESCENT HOME.—Miss Alice A. R. Bunch has been appointed matron. She was trained at Burton-on-Trent General Hospital, and has since been charge sister at Brompton Hospital, at Birmingham General Hospital, at the 1st Southern General Hospital, Birmingham, and assistant matron of St. Mark's Hospital, City Road, E.C.

HEREFORD GENERAL HOSPITAL.—Miss Agnes Gummer has been appointed sister. She was trained at the North Staffs Infirmary, Stoke-on-Trent, where she has since been staff nurse in the $x$-ray department.

ISOLATION HOSPITAL, MERTHYR TYDFIL.—Miss A. Hunt has been appointed matron. She was trained at Walsall Infirmary, has been ward sister at the Bradford Union Hospital, midwife at Firvale, Sheffield, sister at the Borough Hospital, Southampton, assistant matron at the City Hospital, Newcastle-upon-Tyne, and matron of the Pontypridd Isolation Hospital.

KENSINGTON AND FULHAM HOSPITAL, EARLS COURT.—Miss Mary Drury has been appointed out-patient sister. She was trained at Greenwich Infirmary, where she has since been staff nurse and sister. She holds the certificate of the Central Midwives Board.

MOTHERCRAFT TRAINING CENTRE, TREBOVIR ROAD, EARLS COURT.—Miss Townsend has been appointed home sister. She was trained at Royal Infirmary, Bradford, and at the British Hospital for Mothers and Babies, Woolwich, where she obtained the certificate of the Central Midwives Board. She has since been sister at the East End Mothers' Home and of the venereal department at Bradford Royal Infirmary.

PINEWOOD SANATORIUM, WOKINGHAM, BERKS.—Miss Alice Maud Rennie has been appointed matron. She was trained at Greenock Infirmary, has been staff nurse at Nordrach-upon-Mendip Sanatorium, Queen's Nurse at Corstorphine, and matron of the Ochil Hills Sanatorium, Kinross-shire.

QUEEN'S HOSPITAL, BIRMINGHAM.—Miss Hilda Butt has been appointed night sister. She was trained at Gloucester Royal Infirmary, and has since been night sister and theatre sister at the Women's and Children's Hospital, Leeds; theatre sister at the 2nd Southern General Hospital, Bristol, at the No. 6 General Hospital, Rouen, at Casualty Clearing Stations, and at the Children's Hospital, Birmingham.

SHEFFIELD STREET HOSPITAL, KINGSWAY, W.C.—Miss Beatrice Reynolds has been appointed superintendent sister. She was trained at Wandsworth Infirmary, and has since been ward sister and night superintendent.

SHEFFIELD WORKS CONVALESCENT HOME, MATLOCK.—Miss Mary Helen Cotter has been appointed matron. She was trained at Sheffield Royal Infirmary, where she was later sister of the women's medical ward.

WHITEHAVEN AND WEST CUMBERLAND INFIRMARY.—Miss Emily Kay has been appointed matron. She was trained at the General Infirmary, Leeds, has been sister of the women's ward at the Royal Hospital, Richmond, casualty and out-patient sister at the Royal Hospital, Portsmouth, and assistant matron at the General Infirmary, Kilmarnock.

### The College of Nursing
### (Limited by Guarantee).

BIRMINGHAM THREE COUNTIES CENTRE.

**Tuesday, June 22, 5 p.m.**—General meeting (members only) in the Lecture Theatre of the General Hospital, Birmingham. Mrs. Ring (Secretary to the Birmingham Society for Equal Citizenship) has kindly consented to give an address on her experiences in Austria and her visits to the Austrian hospitals. Non-members are cordially invited. Entrance free.

BRIGHTON AND HOVE CENTRE.

IN connection with this Centre a picnic to Pyecombe has been arranged for Saturday, June 19, and it is hoped that as many members as possible will come. Those going by 'bus should meet at the Aquarium at 2 P.M., and those cycling or going by any other means are asked to be in front of the Plough Inn at Pyecombe at 3.15 P.M. Members are to provide their own refreshments, and are asked to bring a Thermos flask if they have one.

The Hon. Secretary will be glad to receive a postcard from each member who proposes to go, who is asked to state means of travelling.

Members are further advised that a Jumble Sale has been arranged for Wednesday, June 30, and their help in securing contributions, in arranging at 2 P.M. on the 29th, and at the Sale at 2 P.M. on the 30th will be gratefully welcomed. The Sale will be held at St. Anne's Hall, St. George's Road, Kemp Town. The Hon. Secretary asks for a postcard from those willing to help.

### The Incorporated Society of Trained Masseuses.

PROGRAMME OF MEMBERS' CONFERENCE, JUNE 24-26, 1920.

THURSDAY, June 24.—11 A.M.—Address by Professor Arthur Keith, on "The Art of Healing," at the Mortimer Hall, 93 Mortimer Street, W. 1.

3 P.M.—Demonstrations on (1) "General Massage," by Miss B. M. G. Copestake; (2) "Some Conditions Occurring in the Shoulder Joint and their Treatment by Massage and Exercises," by Miss Ida Shires. At King's College Hospital, Denmark Hill, S.E. Electric trains Victoria to Denmark Hill; Trams, No. 58 from Victoria, No. 56 from Embankment, and 'Bus No. 68 from Southampton Row or Kingsway.

6 P.M.—Lecture (with lantern illustrations) by Sir Charles Ballance, K.C.M.G., C.B., on "The Healing Process in Nerves," at the Royal Society of Arts, 18 John Street, Adelphi, W.C. 2.

FRIDAY, JUNE 25.—11 A.M.—Lecture by H. Chapple, F.R.C.S., on "The Necessity of Massage and Exercises in the Puerperium," at the Mortimer Hall, 93 Mortimer Street, W. 1.

3 P.M.—Demonstrations of Educational Gymnastics, Dancing, etc., at the Chelsea College of Physical Education, Manresa Road, S.W. (Omnibus 19 or 22 from Piccadilly Circus to Chelsea Town Hall, or District Railway to South Kensington Station.)

6.30 P.M.—Lecture (with lantern illustrations) by Miss Muriel Bond, B.Sc., on "Some Factors Influencing Nutrition and Growth," at the Royal Society of Arts.

SATURDAY, JUNE 26.—10.30 A.M.—Demonstration of "Crawling Exercises for Scoliosis," by E. B. Clayton, M.B., B.C. (Director of the School of Massage, S.R.E., and Medical Electricity, King's College Hospital, Denmark Hill, S.E.), at King's College Hospital (see above), followed at 11.30 A.M. by a Discussion.

Tickets, duly filled in, *must* be produced for admission to any part of the Conference. Season tickets (transferable) for whole Conference, 7s. 6d. Apply to the Secretary, 157 Great Portland Street, W.

